# 

**Published:** 1894-02-24

**Authors:** 


### CONTENTS.

**Is Darwinism a Dying Faith?** - - - - - - - - 363

**Around the Hospitals** - - - - - - - - - 364

**On Modern Progress in Ophthalmic Medicine and Surgery.** By ROBERT BRUDENELL CARTER, F.R.C.S. - - 365

**Art and Disease.** VI.—Healing of the Sick - - - - - - 366

**The Study of Man.** V.—The Minds of Nations - - - - 367

**The Field of Science** - - - - - - - - - 368

**Medical Progress and Hospital Clinics—**

LONDON HOSPITAL.—The Preparation of Patients for Operation 371

Cancer of the Cervix Uteri - - - - - - - - - 372

MEDICAL PROGRESS—Diseases of the Throat - - - - - 373

PROGRESS IN SURGERY - - - - - - - - - 376

NEW APPLIANCES AND THINGS MEDICAL - - - - - 378

**Annotations.**—Henry VIII. to the Rescue—Our Failing Teeth—Evening Out-Patients - - - - - - - - - 369

**Notes and News** - - - - - - - - - - 370

**The Institutional Workshop—**

WITHIN THE HOSPITALS—Three Provincial Hospitals - - - 379

HOSPITAL FINANCE. III.—Voluntary Support - - - - 380

PRACTICAL DEPARTMENTS—The Red Cross Ambulance - - - 381

**Special Appeals.**—The Shipwrecked Fishermen and Mariners' Benevolent Society - - - - - - - - - 382

**Editor's Letter Box.**—Art and Disease: A Solution Offered—The Opium Problem - - - - - - - - - 382

**Hospital Meetings** - - - - - - - - - 383

**The Book World of Medicine and Science** - - - - 383

### EXTRA SUPPLEMENT.—THE NURSING MIRROR.

**News from the Nursing World.**—A Royal Patient—The Hilda May Cot—Another New Club—Nursing at Mortlake—Sure and Simple—Hospital Progress—A Pleasant Entertainment—Am I My Brother's Keeper?—A Nurse's First Duty—Friendly Sailors—On Duty Night and Day—Queen's Nurses in Wales—Aberdeen—Parisian Precautions—Comfortable Complacency—Short Items - - - - cci

**On Nursing the Nervous and Insane.** VII.—Dementia or Mental Enfeeblement - - - - - - - - cciii

**Lectures at St. George's Hospital.**—The History of the Hospital - - - - - - - - - - ccv

**District Nursing.**—VIII. Maternity Work (*continued*) - - cciv

**Old-Time Nurses.** II.—A Brave Life - - - - - - ccvi

**Trained Nurses at Bangor—News from Toronto** - ccvii

**A Lecture on Cholera** - - - - - - - - ccvii

**Professor Horsley on Trephining in the Stone Age** - ccviii

**For Reading to the Sick** - - - - - - - ccviii

**The Muses' Looking Glass.**—"Dick Sheridan," Comedy [illegible] Theatre - - - - - - - - - - - ccix

**Women's Volunteer Medical Staff Corps** - - - - ccix

**Notes and Queries** - - - - - - - - - ccix

**Everybody's Opinion** - - - - - - - - - ccx

